# Supplementary material for: Yellow Pigment Powders Based on Lead and Antimony: Particle Size and Colour Hue
Source: J Imaging. 2021 Jul 30;7(8):127. doi: 10.3390/jimaging7080127 (PMC8404923; doi:10.3390/jimaging7080127)
Supplement: Supplementary file 1 [file jimaging-07-00127-s001.zip › jimaging-1297153-supplementary.pdf]

Journal of Imaging

Article

Yellow Pigment Powders Based on Lead and Antimony: Particle Size and Colour Hue

Giuseppe Capobianco [1], Giorgia Agresti [2], Giuseppe Bonifazi [1], Silvia Serranti [1] and Claudia Pelosi [2,*]

[1] Department of Chemical Engineering Materials & Environment, Sapienza, Rome University, Via Eudossiana 18, 00184 Rome, Italy; giuseppe.capobianco@uniroma1.it (G.C.); giuseppe.bonifazi@uniroma1.it (G.B.); silvia.serranti@uniroma1.it (S.S.)
[2] Department of Economics, Engineering, Society and Business Organization, Laboratory of Diagnostics and Materials Science, University of Tuscia, Largo dell'Università, 01100 Viterbo, Italy; agresti@unitus.it
* Correspondence: pelosi@unitus.it; Tel.: +39-0761-357673

Method

X-ray diffraction (XRD) analysis was performed on samples APB1, APB2 and APB3 through a Siemens Kristalloflex Diffractometer equipped with a Cu anode (λ=1.5418 Å) operating at 40 kV (accelerating voltage) and 30 mA (beam current).

Results

XRD data are reported in the Tables S1-S3. FWHM conventionally is the Full Width Half Maximum of the diffraction peaks and gives a measure of the energy resolution.

Table S1. XRD data for sample APB1.

2-Theta	d(Å)	Height	I/I_0 (%)	Area	FWHM
14.936	5.93	383	3	2254	0.075
27.039	3.30	2962	24	47208	0.141
28.677	3.11	240	2	2222	0.082
**29.980**	**2.98**	**12557**	100	**207215**	**0.146**
31.285	2.86	999	8	14308	0.127
34.734	2.58	3991	32	71588	0.159
44.755	2.02	1108	9	19488	0.156
49.825	1.83	4162	33	89768	0.191
52.989	1.73	959	8	18021	0.167
55.528	1.65	268	2	5358	0.177
59.155	1.56	3343	27	84832	0.225
62.050	1.49	940	7	22494	0.212
65.047	1.43	146	1	1875	0.114

Table S2. XRD data for sample APB2.

2-Theta	d(Å)	Height	I/I_0 (%)	Area	FWHM
14.968	5.91	372	3	3611	0.086
27.053	3.29	3057	26	41842	0.122
28.691	3.11	180	2	2067	0.101
**29.980**	**2.98**	**11941**	100	**178437**	**0.133**
31.299	2.86	1061	9	14240	0.119
34.720	2.58	3844	32	61053	0.140

42.788	2.11	127	1	2551	0.178
44.742	2.02	1245	10	17010	0.121
45.555	1.99	132	1	1039	0.069
49.810	1.83	4379	37	82021	0.166
52.989	1.73	1088	9	17287	0.141
55.540	1.65	324	3	3428	0.094
59.140	1.56	3855	32	79652	0.183
62.006	1.50	965	8	21661	0.199
65.066	1.43	150	1	2267	0.134

Table S3. XRD data for sample APB3.

2-Theta	d(Å)	Height	I/I_0 (%)	Area	FWHM
14.994	5.90	286	3	3369	0.104
27.040	3.29	2863	25	37122	0.115
28.677	3.11	164	1	1694	0.092
**29.994**	**2.98**	**11362**	100	**153625**	**0.119**
31.299	2.86	1014	9	14865	0.130
34.721	2.58	4146	36	57362	0.122
42.834	2.11	122	1	1804	0.131
44.755	2.02	1310	12	15154	0.103
49.810	1.83	4793	42	74764	0.138
52.975	1.73	1270	11	16690	0.117
55.565	1.65	337	3	3931	0.103
59.125	1.56	4434	39	76255	0.152
62.034	1.49	1104	10	21818	0.175
65.036	1.43	240	2	2159	0.080

Discussion

The XRD patterns of samples APB1, APB2 and APB3 exhibit the main d(Å) values at 3.29, 2.98 (100%), 2.58, 1.83, and 1.56.

The research in XRD databases did not find any correspondence with known antimonates, such as $Pb_2Sb_2O_7$ (JCPDS file 18-687), $Pb_{2.5}Sb_{1.5}O_{6.75}$ (JCPDS file 43-0883), $Pb_{3+x}Sb_2O_{8+x}$ (JCPDS file 34-1196), and $PbSb_2O_6$ (JCPDS file 34-0912), this last being the compound named rosiaite often found in combination with $Pb_2Sb_2O_7$ also in artworks [18].

All these compounds were found in yellow samples prepared without the addition of NaCl or $C_4H_5KO_6$.

As a consequence of this result, we may suppose that the addition of sodium chloride caused the production of Pb/Sb compounds incorporating also Na and Cl whose composition is not known or no XRD pattern is present in the JCPDS database.
